# A Nomogram Based on Combining Clinical Features and Contrast Enhanced Ultrasound LI-RADS Improves Prediction of Microvascular Invasion in Hepatocellular Carcinoma

**DOI:** 10.3389/fonc.2021.699290

**Published:** 2021-07-08

**Authors:** Hang Zhou, Jiawei Sun, Tao Jiang, Jiaqi Wu, Qunying Li, Chao Zhang, Ying Zhang, Jing Cao, Yu Sun, Yifan Jiang, Yajing Liu, Xianli Zhou, Pintong Huang

**Affiliations:** ^1^ Department of Ultrasound in Medicine, The Second Affiliated Hospital of Zhejiang University School of Medicine, Hangzhou, China; ^2^ Department of In-Patient Ultrasound, The Second Affiliated Hospital of Harbin Medical University, Harbin, China

**Keywords:** hepatocellular carcinoma, nomogram, liver imaging and reporting and data system, contrast enhanced ultrasound, microvascular invasion

## Abstract

**Purposes:**

To establish a predictive model incorporating clinical features and contrast enhanced ultrasound liver imaging and reporting and data system (CEUS LI-RADS) for estimation of microvascular invasion (MVI) in hepatocellular carcinoma (HCC) patients.

**Methods:**

In the retrospective study, 127 HCC patients from two hospitals were allocated as training cohort (n=98) and test cohorts (n=29) based on cutoff time-point, June 2020. Multivariate regression analysis was performed to identify independent indicators for developing predictive nomogram models. The area under receiver operating characteristic (AUC) curve was also determined to establish the diagnostic performance of different predictive models. Corresponding sensitivities and specificities of different models at the cutoff nomogram value were compared.

**Results:**

In the training cohort, clinical information (larger tumor size, higher AFP level) and CEUS LR-M were significantly correlated with the presence of MVI (all p<0.05). By incorporating clinical information and CEUS LR-M, the predictive model (LR-M+Clin) achieved a desirable diagnostic performance (AUC=0.80 and 0.84) in both cohorts at nomogram cutoff score value of 89. The sensitivity of LR-M+Clin when predicting MVI in HCC patients was higher than that of the clinical model alone (86.7% vs. 46.7%, p=0.027), while specificities were 78.6% and 85.7% (p=0.06), respectively, in the test cohort. In addition, LR-M+Clin exhibited similar AUC and specificity, but a significantly higher sensitivity (86.7%) than those of LR-M alone and LR-5(No)+Clin (both sensitivities=73.3%, both p=0.048).

**Conclusion:**

The predictive model incorporating CEUS LR-M and clinical features was able to predict the MVI status of HCC and is a potential reliable preoperative tool for informing treatment.

## Introduction

Although liver resection and transplantation are the first-line therapeutic options for hepatocellular carcinoma (HCC), they are still associated with low post-operative 5-year recurrence-free survival (as low as 26-40%) (1, 2). Microvascular invasion (MVI) is one of the most useful prognostic parameters for predicting the survival rate of HCC patients (3). As previously reported (4), compared to MVI negative patients, MVI positive patients are more likely to develop intrahepatic metastasis, leading to unsatisfactory overall survival outcomes. Thus, a wider range of surgical margin (>5mm) is preferred for HCC with positive MVI status (5). However, currently, microscopic evaluation of post-operative tumor specimens is the only available strategy for determining MVI status in HCC. Therefore, it is important to establish a predictive model, based on pre-surgical variables, to predict the MVI status, which could be important in informing treatment.

Studies have evaluated the relationships between MVI and preoperative demographic information, serum and cancer biomarkers, but they have reported contrasting findings (6–8). In addition, features on contrast enhanced imaging modalities are significant tools for characterizing focal liver lesions (9). When comparing with contrast enhanced computed tomography (CECT) and contrast enhanced magnetic resonance imaging (CEMRI), contrast enhanced ultrasound (CEUS) enables real-time scanning by injecting blood-pool agents (10) and truly reflects the vascular condition within tumor microenvironment with great convenience and cost-effectiveness (11). CEUS perfusion characteristics [i.e. patterns of arterial phase hyperenhancement (APHE) and washout (WO)] can be used as bio-signatures for identifying aggressive biological behavior, such as tumor differentiation grade (12, 13) and MVI pattern (14–16). However the above mentioned studies were all carried out in single center. Moreover, variable definitions of imaging features in these studies inhibit their further clinical applications.

With the aim of improving standard data collection, interpretation and communications among medical institutions, the American College of Radiology (ACR) released several editions of liver imaging reporting and data system (LI-RADS) based on CEUS and CECT/CEMRI. Interestingly, CEMRI LR-M can accurately distinguish between HCC and other malignancies (17), and is also associated with worse prognosis (18). However, to the best of our knowledge, studies have not yet evaluated the prognostic values of preoperative CEUS LI-RADS classifications in estimating MVI patterns among HCC patients.

Hence, we aimed at establishing a predictive model incorporating clinical and CEUS features for determining MVI status among HCC patients.

## Materials and Methods

This retrospective study was approved by the ethical committees of two hospitals with a waiver of patient’s informed consent. But all patients signed written informed consents before CEUS examination and surgery.

### Patient Selection

**Figure 1:**
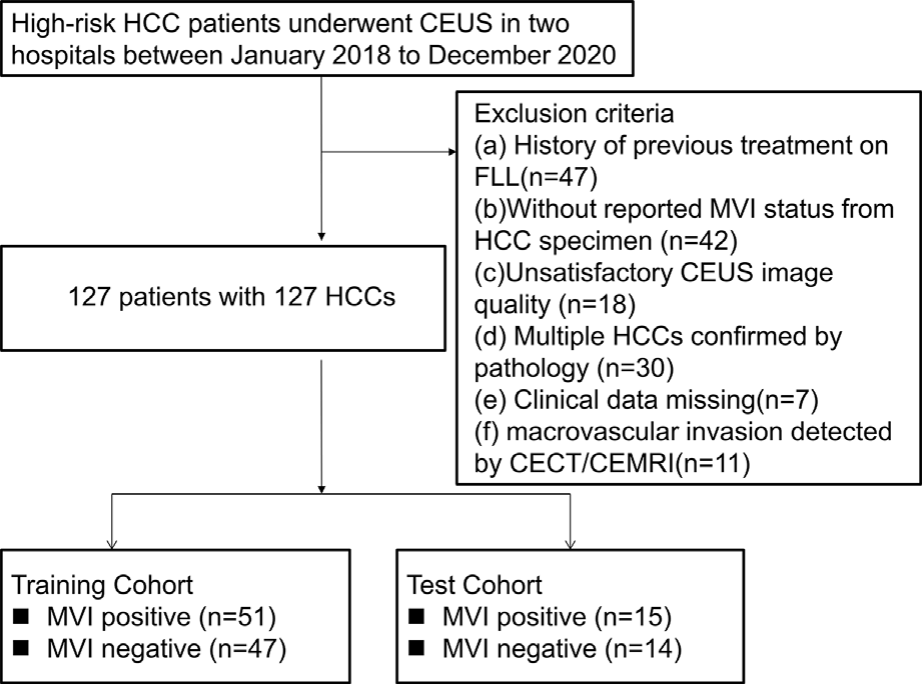
Flowchart of patient selection. HCC, hepatocellular carcinoma; MVI, microvascular invasion; CEUS; contrast enhanced ultrasound; CECT, contrast enhanced computed tomography; CEMRI, contrast enhanced magnetic resonance imaging; FLL, focal liver lesion.

Per recommendations of the ACR group, the study population only consisted of high-risk HCC patients, including hepatitis B infection, cirrhosis and previous history of HCC (19). The exclusion criteria were: (i). A history of previous treatment on FLL, such as ablation, chemotherapy, immunotherapy; (ii). Those without reported MVI status from HCC specimens; (iii). Unsatisfactory CEUS image quality; iv. Multiple pathologically confirmed HCCs; (v). Missing clinical data; and (vi). Macrovascular invasion detected by imaging. From January 2018 to December 2020, a total of 127 HCC patients were enrolled from the two hospitals. Based on the cutoff time-point, June 2020, HCC patients in the preceding period were defined as the development cohort for establishing the nomogram model (n=98). The other 29 HCC patients were included as the external test cohort (**Figure 1**).

### Clinical Information

Tumor size was determined by US imaging. Preoperative serum examination included liver and renal function, hepatitis B and C immunology, α-fetoprotein level (AFP) and coagulation function tests. These indices were measured before surgery within 2 weeks.

### US and CEUS Examination

Liver US and CEUS examinations were performed by six experienced radiologists from two centers. All examinations were carried out on ESAOTE (MyLab 90 X-vision, Italy), Philips IU22 (Philips Medical Solutions; Mountain View, CA, United States), Aplio 500 (Toshiba Medical Systems, Tokyo, Japan), Aixplorer Ultrasound system (SuperSonic Imagine, AixenProvence, France) and Resona 7 (Mindray, Shenzhen, China). Before CEUS, all patients were subjected to routine liver US examination. Entire liver sections were scanned with conventional US. When the target lesion was found, the largest cross-section of tumor diameter was examined and recorded. Then the CEUS was initiated. After a bolus injection of 2.4mL SonoVue (Bracco, Milan, Italy) through peripheral venous cannula, a 5-mL saline flush was following and the timer on the sonography was started. The second CEUS examination would be repeated 15 minutes later If the image quality during first injection was unsatisfactory (n = 5). The FLLs were continuously scanned in the first 60 seconds to capture the arterial phase and then observed each intermittently for 20–30 seconds for image requisition until the microbubbles cleared from circulation (usually up to 5 minutes). All video clips were recorded and then transferred to a hard disk.

### US Image Interpretation

Two experienced liver radiologists (more than 5 years’ experience for both) reviewed US images, who did not take part in data acquisition and were blinded to clinical information and pathological results. In brief, echogenicity was defined as isoechoic, hypoechoic or hyperechoic when comparing with the echogenicity of surrounding parenchyma. The shape of the lesion was determined as round/oval or irregular. Moreover, the margin was categorized as well defined or poorly defined. Halo sign was categorized as presence and absence. Regarding color doppler images, intratumoral vascularity was categorized as absent (1-3 vessels segment within the mass), a few or very rich (more than 3 vessels segment within the mass). In cases of discrepancies, a consensus was achieved.

### CEUS LI-RADS Interpretation

The same two above mentioned liver radiologists evaluated CEUS clips. CEUS definitions of FLL were strictly based on suggestions of CEUS LI-RADS (19), as shown in **Supplemental Table 1**. If there was a discrepancy, a consensus was achieved.

A FLL is defined as LR-3 (a) if it exceeds 20mm, combining no APHE without late and mild washout (WO); (b) or if it (≥10mm) shows APHE, without late and mild WO; (c) or if it (<10mm) shows APHE without late and mild WO.

A FLL is determined as LR-4 (a) if it exceeds 20mm, combining no APHE with late and mild WO; (b) or if it (<20mm) shows no APHE, regardless of late and mild WO presence; (c) or if it (<10mm) shows APHE with late and mild WO.

If a FLL (>10mm) is manifested as arterial phase enhancement (APHE) followed by late and mild washout (WO), it is categorized as LR-5. Furthermore, if a FLL shows rim enhancement, or early WO or marked WO, then it should be categorized as LR-M regardless of lesion size.

### Statistical Analysis

After normal distribution tests, continuous variables were expressed as mean ± standard deviation while categorical variables were expressed as percentages. Continuous data were compared by independent t tests while categorical data were compared using Chi-square tests or Fisher’s tests if necessary. In the training cohort, significant parameters between MVI negative and MVI positive patients were enrolled in the multivariate regression model by stepwise forward selection method. Then, independently significant indicators for MVI positive patterns were used for further predictive model establishment as follows: Model I, nomogram model combining clinical features and CEUS LR-M category (Clin+LR-M); Model II, nomogram model combining clinical features and CEUS LR-5 category absence (Clin+LR-5) and Model III, nomogram model based on clinical features (Clin). The diagnostic performances of the predictive models were tested in both the training and test cohorts. The area under receiver operating characteristics curve (AUC) was established to indicate diagnostic performance of different predictive models, with the cutoff value being the point corresponding to the highest Youden index. Comparisons of AUC were determined using the Delong test, both in the training and test cohorts. Sensitivities, specificities, positive predictive values (PPV) and negative predictive values (NPV) were compared by Chi-square test. Inter-reader agreement was calculated by the intraclass coefficient (ICC) model. Inter-reader agreement was deemed as poor (0-0.2), fair (0.2-0.4), moderate (0.4-0.6), good (0.6-0.8) or excellent (0.8-1.0) (20). Statistical analyses were performed by the SPSS 20.0 software package (Chicago, USA) and Medcalc software (Mariakerke, Belgium). p<0.05 was taken as the threshold for statistical significance.

## Results

### Baseline Characteristics

Incidences of positive MVI patterns were 51.7% and 52.0% in the training and test cohorts, respectively. As shown in **Supplemental Table 2**, there were no significantly different baseline parameters between the training and test cohorts.

**Figure 2:**
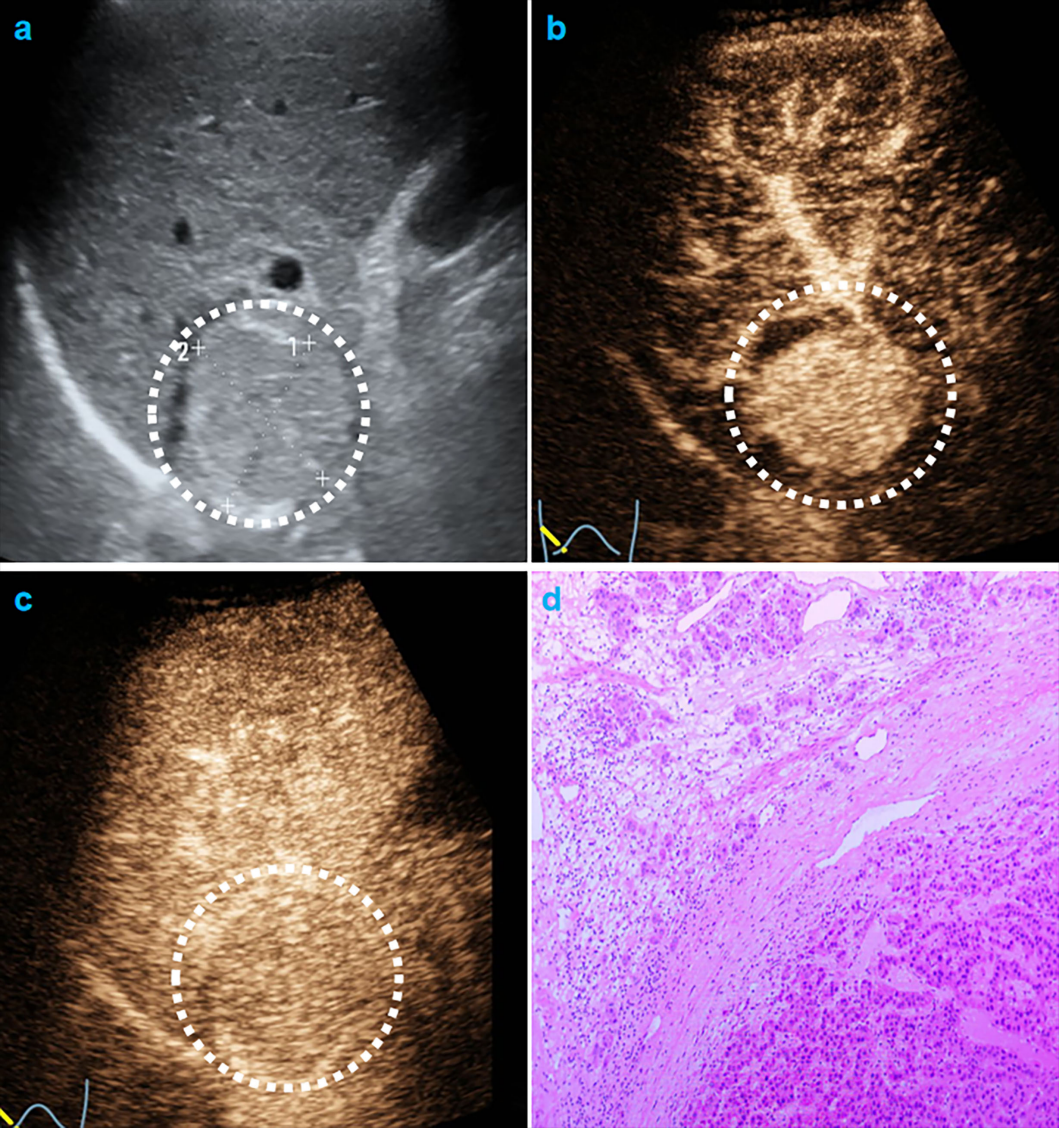
CEUS LR-5 and MVI-negative HCC. Male cirrhotic patient shows a 36.8mm, hypo-echoic HCC (AFP=4.1ng/mL) on US **(A)**. On CEUS, the HCC shows arterial phase hyper-enhancement (25s, **B**) and mild and late washout (80s,**C**). The pathological specimen shows well differentiated HCC and absence of MVI **(D)**.

**Figure 3:**
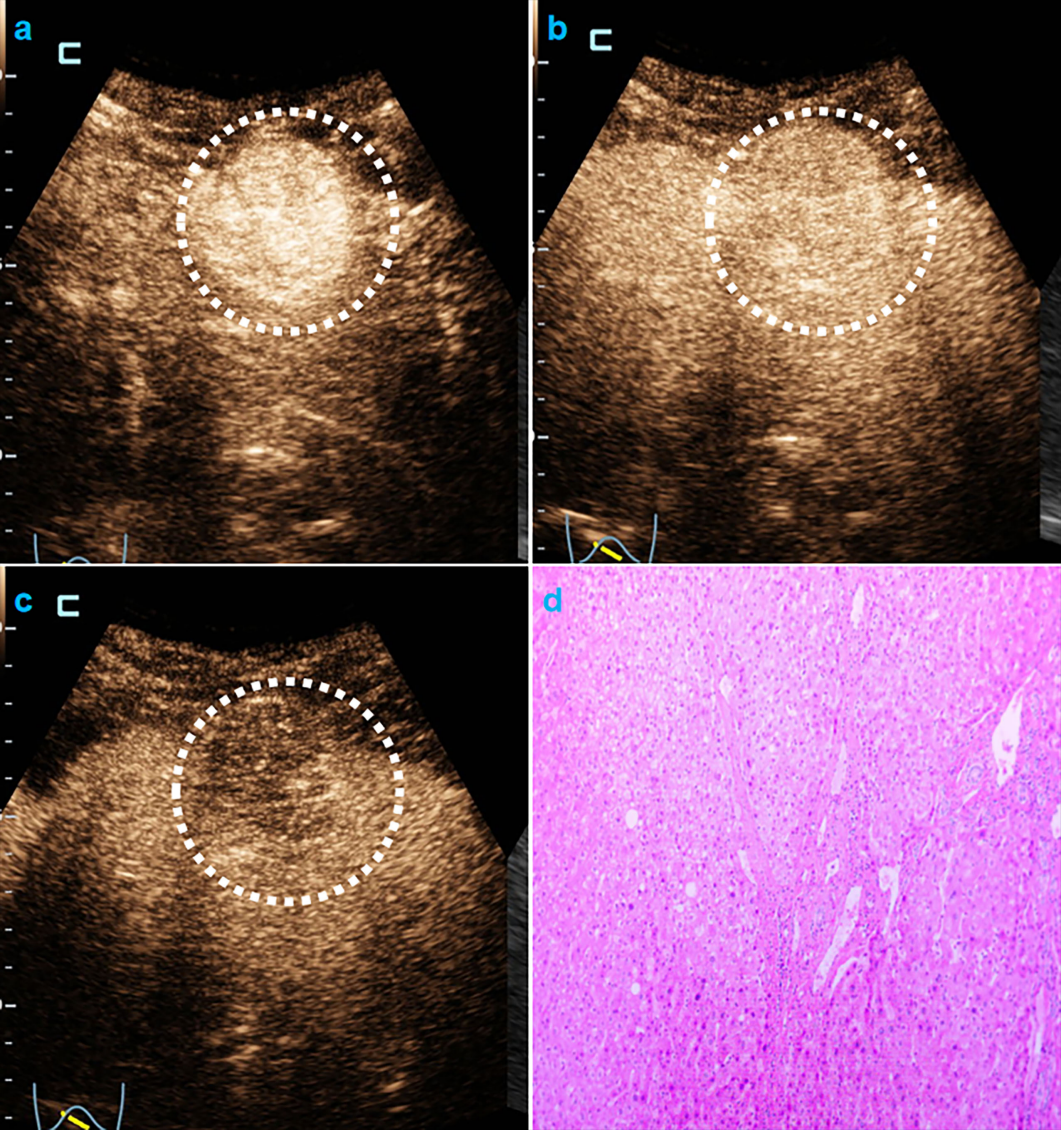
CEUS LR-M and MVI-negative HCC. Male cirrhotic patient shows a 38.4mm HCC (AFP=1.8ng/mL) with arterial phase hyper-enhancement ( 22s, **A**) early (44s, **B**) and mild washout (120s, **C**). The pathological specimen shows well-moderately differentiated HCC and absence of MVI **(D)**.

In univariate analysis (**Table 1**), elevated AFP levels and larger tumor sizes (>30 mm) were found in the MVI positive group (both p<0.05) of the training population. Contrastingly, there were no US features that were significantly associated with MVI patterns (all p>0.05). Regarding CEUS features, late and mild WO, early WO and final CEUS categories were all significant factors for distinguishing the MVI negative group from the MVI positive group (all p<0.05). If LR-M was indicated as an MVI positive predictor, it was significantly different between the two MVI pattern groups (p=0.002). For the test cohort (**Supplemental Table 3**), elevated AFP levels and larger tumor sizes (>30 mm) were more prevalent in the MVI positive population (both p<0.05). Moreover, the late and mild WO plus CEUS LI-RADS category were not indicative of the MVI negative group, whereas early WO and LR-M were significantly correlated with MVI positive patterns (p=0.027). Representative images of CEUS LI-RADS features in predicting MVI are presented in **Figures 2** and **3**.

**Table 1:** Univariate analysis of Clinical, US and CEUS LI-RADS features for predicting MVI status in training cohort.

	MVI Positive (n=51)	MVI Negative (n=47)	P value
**Clinical features**			
Age	58.9 ± 12.2	59.9 ± 10.2	0.458
Male sex(yes)	45 (88.2%)	39 (83.0%)	0.678
Cirrhosis(yes)	40 (78.4%)	41 (87.2%)	0.250
Hepatitis B virus infection(yes)	48 (94.1%)	42 (89.4%)	0.475
PT>14s	14(27.5%)	9(19.1%)	0.352
TT>18s	13(25.5%)	11(23.4%)	1.000
AFP(<20/20-400/>400ng/mL)	21/15/15	35/9/3	**0.002**
Platelet count<100*10^9^	9(17.6%)	12(25.5%)	0.342
Total bilirubin>21umol/L	17(33.3%)	8(17.0%)	0.064
Albumin<35	4(27.5%)	8(17.0%)	0.216
ALT>45U/L	20(39.2%)	18(38.3%)	0.926
AST>35U/L	29(56.9%)	24(54.1%)	0.565
Tumor size>30mm	41 (80.4%)	21 (44.7%)	**<0.001**
**US features**			
Echogenicity(hypo/iso/hyper)	43/4/4	30/7/10	0.062
Poorly defined margin	36 (70.6%)	40 (85.1%)	0.085
Irregular shape	35(68.6%)	37(78.7%)	0.258
Halo sign (yes)	9(17.6%)	16(34.0%)	0.063
Vascularity(no/a few/rich)	21/17/13	20/22/5	0.131
**CEUS LI-RADS**			
CEUS LR-5 major features			
APHE (yes)	45(88.2%)	43(91.5%)	0.743
Late and mild WO (yes)	18(35.3%)	28(59.6%)	**0.016**
CEUS LR-M features			
Rim enhancement (yes)	(3.9%)	2(4.3%)	1.000
Early WO (yes)	28(54.9%)	11(23.4%)	**0.001**
Marked WO (yes)	7(13.7%)	4(8.5%)	0.528
CEUS LI-RADS ancillary features			
Nodule-in-nodule pattern (yes)	7(13.7%)	5(10.6%)	0.641
Mosaic pattern (yes)	9(17.6%)	3(6.4%)	0.125
CEUS LI-RADS category(3/4/5/M)	2/4/15/30	2/3/31/11	**<0.001**
CEUS LR-5 (No)	31(66.0%)	15(29.4%)	**<0.001**
CEUS LR-M (yes)	30(58.8%)	11(23.4%)	**<0.001**

Values in bold mean statistically significant. ALT, Alanine aminotransferase; AST, Aspartate aminotransferase; AFP, alpha fetoprotein; PT, Prothrombin time, TT, thrombin time; CEUS, contrast-enhanced ultrasound; LI-RADS, liver imaging and reporting data system; APHE, arterial phase hyperenhancement; WO, washout.

### Nomogram Model Establishment

**Figure 4:**
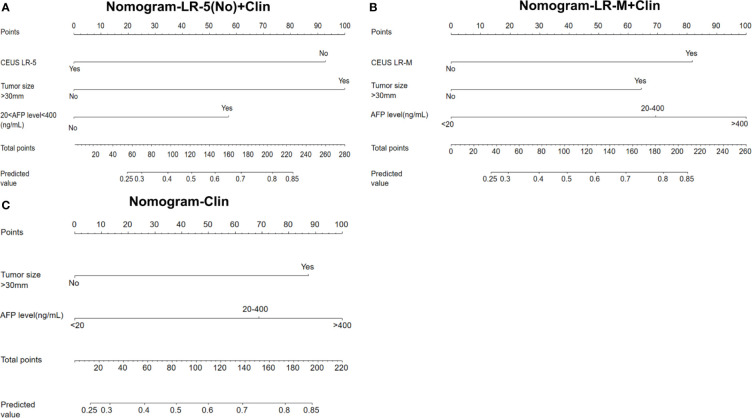
Nomogram graphics of Clin +LR-5(No) model **(A)**, Clin+LR-M model **(B)** and Clin model **(C)** for predicting MVI in HCC patients. Clin, clinical information; HCC, hepatocellular carcinoma; MVI, microvascular invasion; CEUS; contrast enhanced ultrasound; AFP, alpha fetoprotein; LR-5, liver imaging reporting and data system category 5; LR-M, liver imaging reporting and data system category M.

In the multivariate regression model I (**Table 2**), AFP levels higher than 400 ng/mL were found to be the best predictors for positive MVI with an odds ratio (OR) value of 5.12, followed by CEUS LR-M (OR=3.80), 20 ng/mL<AFP level<400 ng/mL (OR=3.11) and tumor size larger than 30 mm (OR=2.86). For Model II, absence of CEUS LR-5 (OR=4.37), 20 ng/mL<AFP level<400 ng/mL (OR=3.27) and tumor size larger than 30 mm (OR=3.74) were significant factors for predicting MVI while AFP levels higher than 400 ng/mL were not. For model III, the clinical nomogram model (namely, Clin) was established using AFP levels and tumor sizes. All nomogram figures are shown in **Figure 4**.

**Table 2:** Multivariate analysis of clinical and CEUS LI-RADS features for predicting MVI in training cohort.

	OR (95% CI)	β value	P value
**CEUS LR-M+Clin**			
20ng/mL<AFP level<400ng/mL	3.11(1.03, 9.34)	1.13	0.044
AFP level >400ng/mL	5.12 (1.18, 22.24)	1.63	0.029
Tumor size>30mm	2.86 (1.02, 8.01)	1.05	0.045
CEUS LR-M(Yes)	3.80(1.44, 10.04)	1.34	0.007
**CEUS LR-5 (No)+Clin**			
20ng/mL<AFP level<400ng/mL	3.27(1.07, 10.05)	1.39	0.038
AFP level >400ng/mL	4.18 (0.96, 18.31)	1.43	0.058
Tumor size>30mm	3.74 (1.33, 10.52)	1.32	0.012
CEUS LR-5(No)	4.37(1.69, 11.33)	1.48	0.002
**Clin**			
20ng/mL<AFP level<400ng/mL	2.91(1.02, 8.30)	1.07	0.031
AFP level >400ng/mL	4.72 (1.15, 19.36)	1.43	0.031
Tumor size>30mm	3.88 (1.46, 10.31)	1.36	0.007

Clin, clinical features; MVI, microvascular invasion; AFP, alpha fetoprotein; CEUS, contrast-enhanced ultrasound; OR, odds ratio.

### Diagnostic Performance of Different Models

**Figure 5:**
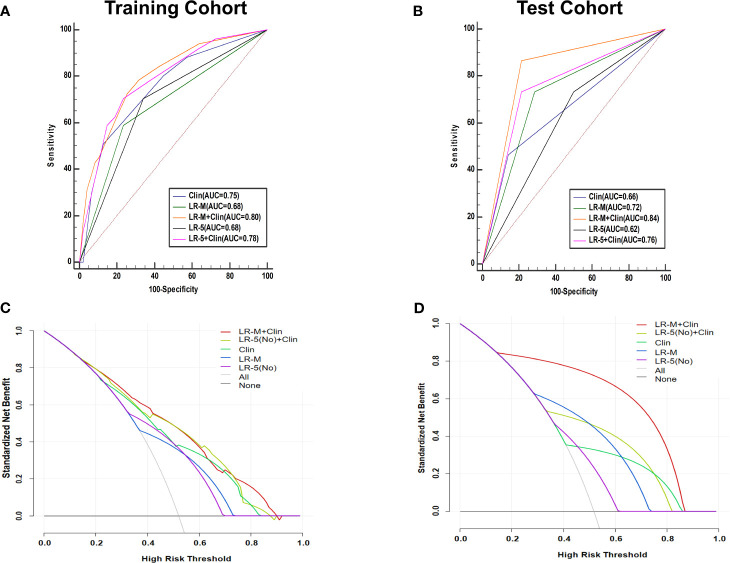
AUC graphics of all models for predicting MVI in HCC patients in the training **(A)** and test cohort **(B)**. Decision curve graphics of all models for predicting MVI in HCC patients in the training **(C)** and test cohort **(D)**. In the decision curve, the y-axis represents net benefit and the x-axis is the value of the different probability, illustrating the trade-offs between benefit (true positives) and harm (false positives) as the threshold probability (preference) is varied across a range of reasonable threshold probabilities. Clin, clinical information; HCC, hepatocellular carcinoma; MVI, microvascular invasion; CEUS; contrast enhanced ultrasound; LR-5, liver imaging reporting and data system category 5; LR-M, liver imaging reporting and data system category M.

At the cutoff value of 100 and 89, respectively, model III achieved an AUC value of 0.75, lower than that of model I in the training cohort (AUC=0.80), but without significant differences (**Table 3** and **Figure 5A**). Besides, model II exhibited a comparable AUC value (0.78) to model I.

**Table 3:** Diagnostic performance of different models for predicting MVI in training and test cohort.

	AUC	Cut-off	Sen (%)	Spe (%)	PPV (%)	NPV (%)
Clin		100				
Training cohort	0.75^^		60.0	87.2	81.2	62.1
	(0.65, 0.83)		(36.6, 68.2)	(74.3, 95.2)	(63.6, 92.8)	(49.3, 73.8)
Test cohort	0.66^&^		46.7*	85.7^	77.8	60.0
	(0.46, 0.83)		(21.3, 73.4)	(57.2, 98.2)	(40.0, 97.2)	(36.1, 80.9)
LR-M		Yes				
Training cohort	0.68^#^		58.8	76.6	73.2	63.2
	(0.58, 0.77)		(44.2, 72.4)	(62.0, 87.7)	(57.1, 85.8)	(49.3, 75.6)
Test cohort	0.72		73.3^$^	71.4	73.3	71.4
	(0.53, 0.87)		(44.9, 92.2)	(41.9, 91.6)	(44.9, 92.2)	(41.9, 91.6)
LR-M+Clin		89				
Training cohort	0.80		72.6	74.5	75.5	71.4
	(0.70, 0.87)		(58.3, 84.1)	(59.7,86.1)	(61.1,86.7)	(56.7, 83.4)
Test cohort	0.84		86.7	78.6	81.3	84.6
	(0.64, 0.94)		(59.5, 98.3)	(49.2, 95.3)	(54.4, 96.0)	(54.6, 98.1)
LR-5(No)		No				
Training cohort	0.68		70.6	66.0	69.2	67.4
	(0.58, 0.77)		(56.2, 82.5)	(50.7, 79.1)	(54.9, 81.3)	(52.0, 80.5)
Test cohort	0.62		73.3^$^	50.0	61.1	63.6
	(0.42, 0.79)		(44.9, 92.2)	(23.0, 77.0)	(35.7, 82.7)	(30.8, 89.1)
LR-5(No)+Clin		150				
Training cohort	0.78		66.7	80.9	79.1	69.1
	(0.69,0.86)		(52.1, 79.2)	(66.7, 90.9)	(64.0, 90.0)	(55.2, 80.9)
Test cohort	0.76		73.3^$^	78.6	78.6	73.3
	(0.57,0.90)		(44.9, 92.2)	(49.2, 95.3)	(49.2, 95.3)	(44.9, 92.2)

data are 95% confidence intervals in parentheses. Clin, clinical features; AUC, area under receiver operating characteristics; Sen, sensitivity; Spe, specificity; PPV, positive predictive value; NPV, negative predictive value.

^&^indicates a significant difference compared with that of LR-M+Clin in the test cohort, P=0.023.

*indicates a significant difference compared with that of LR-M+Clin in the test cohort, P=0.027.

^^^indicates NO significant difference compared with that of LR-M+Clin in the test cohort, P=0.06.

^^indicates NO significant difference compared with that of LR-M+Clin in the training cohort, P=0.19.

^$^indicates a significant difference compared with that of LR-M+Clin in the test cohort, P=0.048.

^#^indicates a significant difference compared with that of LR-M+Clin in the training cohort, P=0.003.

**Figure 6:**
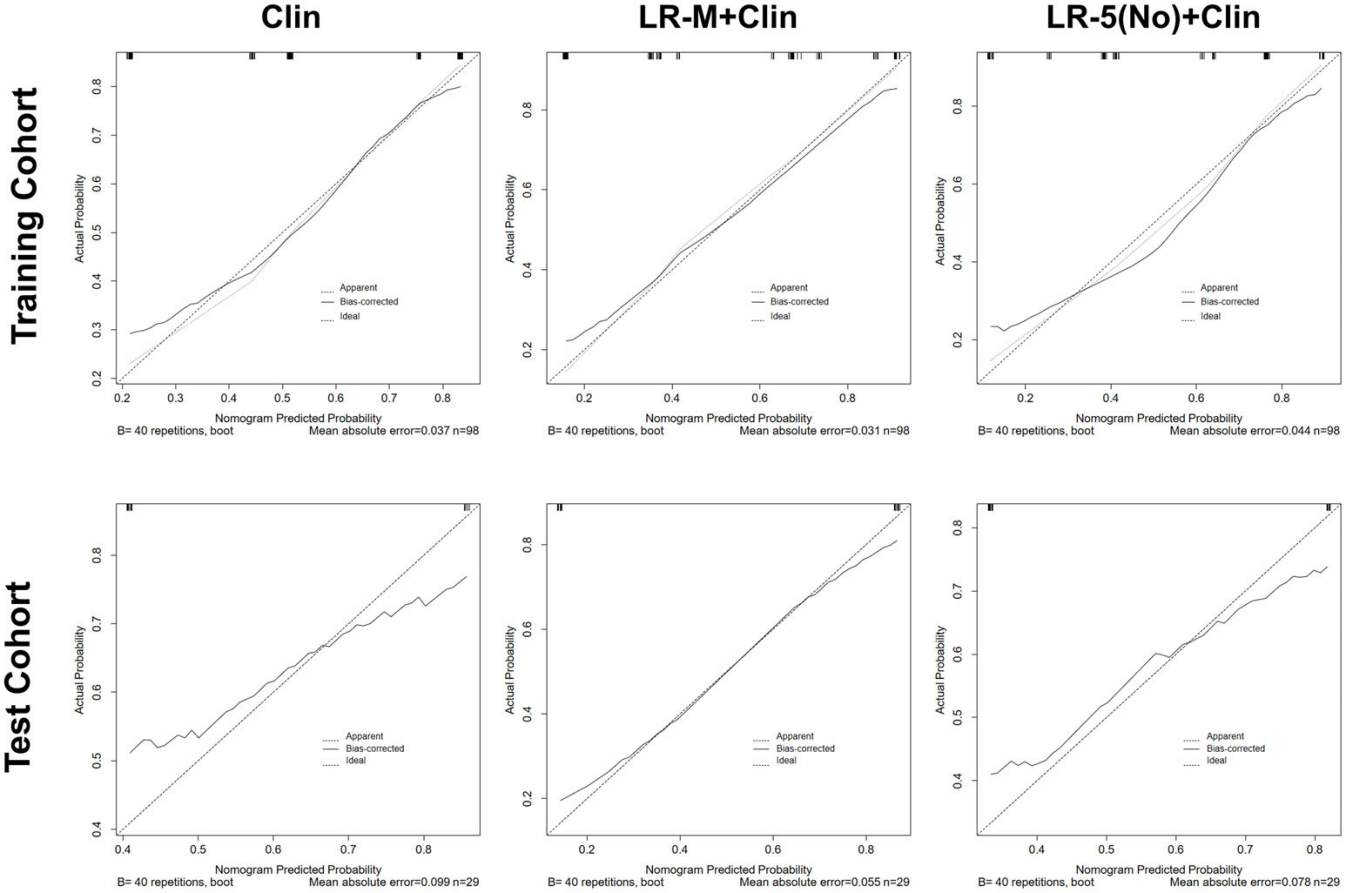
Calibration curve graphics of all models for predicting MVI in HCC patients in the training cohort (upper panel) and test cohort(lower panel). In the calibration curve, X-axis means plotting the predicted probabilities from the model and y-axis means actual survival probabilities. Clin, clinical information; HCC, hepatocellular carcinoma; MVI, microvascular invasion; CEUS; contrast enhanced ultrasound; LR-5, liver imaging reporting and data system category 5; LR-M, liver imaging reporting and data system category M.

In the test cohort, model I achieved a significantly higher AUC value (**Figure 5B**) when compared to that of model III (0.84 vs. 0.66, p=0.023). In addition, the sensitivity of Clin+LR-M was higher than that of Clin model (86.7% vs. 46.7%, p=0.027) without compromising the specificity value (78.6% vs. 85.7%, p=0.06) (**Table 3**). Moreover, model I exhibited similar AUC and specificity, but significantly higher sensitivity (86.7%) than those of LR-M alone and model II (both sensitivities = 73.3%, p=0.048) (**Table 3**). Decision curve graphics (**Figures 5C, D**) revealed a high net benefit of Clin+LR-M model in estimating the MVI risk. Moreover, calibration curves in **Figure 6** show that the Clin+LR-M model, either applied in the training or test cohort, exhibited a good agreement between MVI prediction and final pathological confirmation on the specimen.

### Inter-Reader Agreement

The results showed that inter-reader agreement on CEUS LI-RADS category was good with an ICC value of 0.637[95% confidence interval (CI): 0.503-0.741]. Concerning LR-M category, the ICC value was 0.686 (95% confidence interval: 0.564-0.778), indicating good consistency.

## Discussion

Tumor sizes, AFP levels and CEUS LR-M were found to be significant independent predictors of MVI in HCC patients. We further developed predictive models, consisting of clinical and CEUS features, which are easy to use and can inform management-related decision making.

The role of preoperative clinical information in estimating MVI is conflicting. For example, tumor sizes have been considered to be associated with MVI (21, 22) whereas Suh et al’s findings did not support this conclusion (23). In the present study, tumor sizes larger than 30mm were found to be independent predictors for predicting high probability of MVI, which agrees with previous results (24). It is reasonable to speculate that along with increasing size of tumor diameter, tumor cells invade into adjacent parenchyma and had a higher chance of infiltrating into microvessels. On the other hand, higher AFP levels also are helpful for indicating probability of diagnosing MVI (8, 25). Our results revealed that AFP>400ng/mL was the best predictor for estimating presence of MVI. However, incorporation of these two features in a combined Clin model resulted in a poor diagnostic performance (below 0.70) in predicting MVI with a low sensitivity (below 50%) in the test cohort.

Apart from its diagnostic purpose, contrast enhanced imaging can also be used to identify aggressive behaviors of HCC. Dong et al. (15) showed that on CEUS, wash-in area under the curve and wash-out area under the curve generated from time intensity curves were significantly higher in MVI positive group than in MVI negative group in the center HCC lesions. The current study is the first one to confirm the prognostic potential of CEUS LR-M, a well-established standard algorithm, in predicting MVI in HCC patients.

LR-M was initially designed to encompass various non-HCC malignancies, which exhibited distinct imaging characteristics. However, clinically, there are atypical HCCs that are categorized as LR-M (20). As key LR-M features of CEMRI, rim enhancement and progressive central enhancement (26, 27) were reported to be significant predictors for MVI. Therefore we hypothesized that HCCs classified as CEUS LR-M may also be more inclined to display MVI pattern. As LR-M criteria, early WO was a significant indicator for estimating MVI both in the univariate and multivariate analysis. Zhu et al. (14) suggested that 10.5% (2/19) of HCC patients with no WO had MVI, compared with 86.7% (26/30) with rapid WO (shorter than 30s) tumors (p<0.001). The exact mechanism involving the relationship between early WO and MVI has not been established. A possible explanation is that, assuming the portal vein to be the main drainage vessel of HCC, vascular occlusion caused by tumor thrombi in the minute branch would further decrease vein flow and may thus, contribute to quick WO patterns in the very early portal phase. In addition, larger tumors might exhibit lower degree of differentiation status. The multiplication rate of tumor cells is about two to three times that of endothelial cells and these differences cause further decreased microvascular density with increasing tumor size (28).

By adding CEUS LR-M into Clin model, the LR-M+Clin model presented better AUC and sensitivity than those of Clin model in the test cohort. Most importantly, previous research groups failed to prove the utility of MVI predictive models in multicenter studies (29, 30), hindering the generalization of findings used among different institutions. In the test cohort, the LR-M+Clin model exhibited a higher diagnostic performance than that of the Clin model alone (0.84 vs. 0.66, p=0.023). Using the nomogram score of 89 as the cut-off value, sensitivity of the LR-M+Clin model corresponded to 86.7%, higher than that of the Clin model (cutoff value: 100; sensitivity: 46.7%, p=0.027) without losing specificity (78.6% vs. 85.7%, p=0.06). In addition, the LR-M+Clin model exhibited comparable AUC and specificity, but a significantly higher sensitivity (86.7%) than those of the LR-M alone and model II (both sensitivities =73.3%, p=0.048), further validating the diagnostic power of combining CEUS LR-M and clinical information.

This study had several limitations. First, its retrospective nature may have led to inevitable selection bias. Therefore, a prospective study is required to verify our proposed predictive model. Second, our sample size is relatively small, especially in the test cohort. Third, different US machines were used to collect CEUS data, which may result in image variability. The limited number of patients in the test cohort inhibits subgroup evaluation of US-machine-derived inconsistencies. Prospective large sample-size studies can resolve this problem. Fourth, for multiple hepatic lesions, we selected the largest one on US for CEUS examination, due to limited acoustic window (31). However, the selection criteria may have led to greater bias, especially given that multiplicity is also a predictor for MVI in HCC patients (8). Fourth, quantitative analysis of interpreting CEUS features would be much better and urgently needed to address the inconsistency involving the naked-eye observation. Finally, we did not compare the diagnostic performance of CEUS LI-RADS versus CECT/CEMRI LI-RADS in predicting MVI and further study is needed in the near future.

In conclusion, clinical information (larger tumor size, higher AFP level) and CEUS LR-M are significantly correlated with the presence of MVI. By incorporating clinical information and CEUS LR-M, the predictive model achieved a good diagnostic performance and a high sensitivity for predicting MVI in HCC patients.

## Data Availability Statement

The original contributions presented in the study are included in the article/**Supplementary Material**. Further inquiries can be directed to the corresponding authors.

## Ethics Statement

The studies involving human participants were reviewed and approved by The second affiliated hospital of Zhejiang University and The second affiliated hospital of Harbin Medical University. The ethics committee waived the requirement of written informed consent for participation.

## Author Contributions

Conception and design of the study: PH and XZ. Ultrasound data acquisition: CZ, JS, PH, and XZ. Clinical and pathological data collection: YL, JC, YS, and YJ. Analysis and interpretation of data: QL, JW, HZ, TJ, and YZ. Drafting the manuscript: HZ. Revising and final approval of the version to be published: PH. All authors contributed to the article and approved the submitted version.

## Funding

The study was supported by the National Key R&D Program of China (2018YFC0115900), National Natural Science Foundation of China (Grant No. 82030048, 81527803, 81901871, 82001818), and Natural Science Foundation of Zhejiang Province (LQ20H180009; LQ21H180007).

## Conflict of Interest

The authors declare that the research was conducted in the absence of any commercial or financial relationships that could be construed as a potential conflict of interest.

## Abbreviations

CEUS, Contrast-enhanced ultrasound; LI-RADS Liver imaging and reporting and data system; HCC, Hepatocellular carcinoma; MVI, Microvascular invasion; APHE, Arterial phase hyperenhancement; WO, Washout.
