# Supplementary figures and images for: A Nomogram Based on Combining Clinical Features and Contrast Enhanced Ultrasound LI-RADS Improves Prediction of Microvascular Invasion in Hepatocellular Carcinoma
Source: Front Oncol. 2021 Jul 8;11:699290. doi: 10.3389/fonc.2021.699290 (PMC8297520; doi:10.3389/fonc.2021.699290)

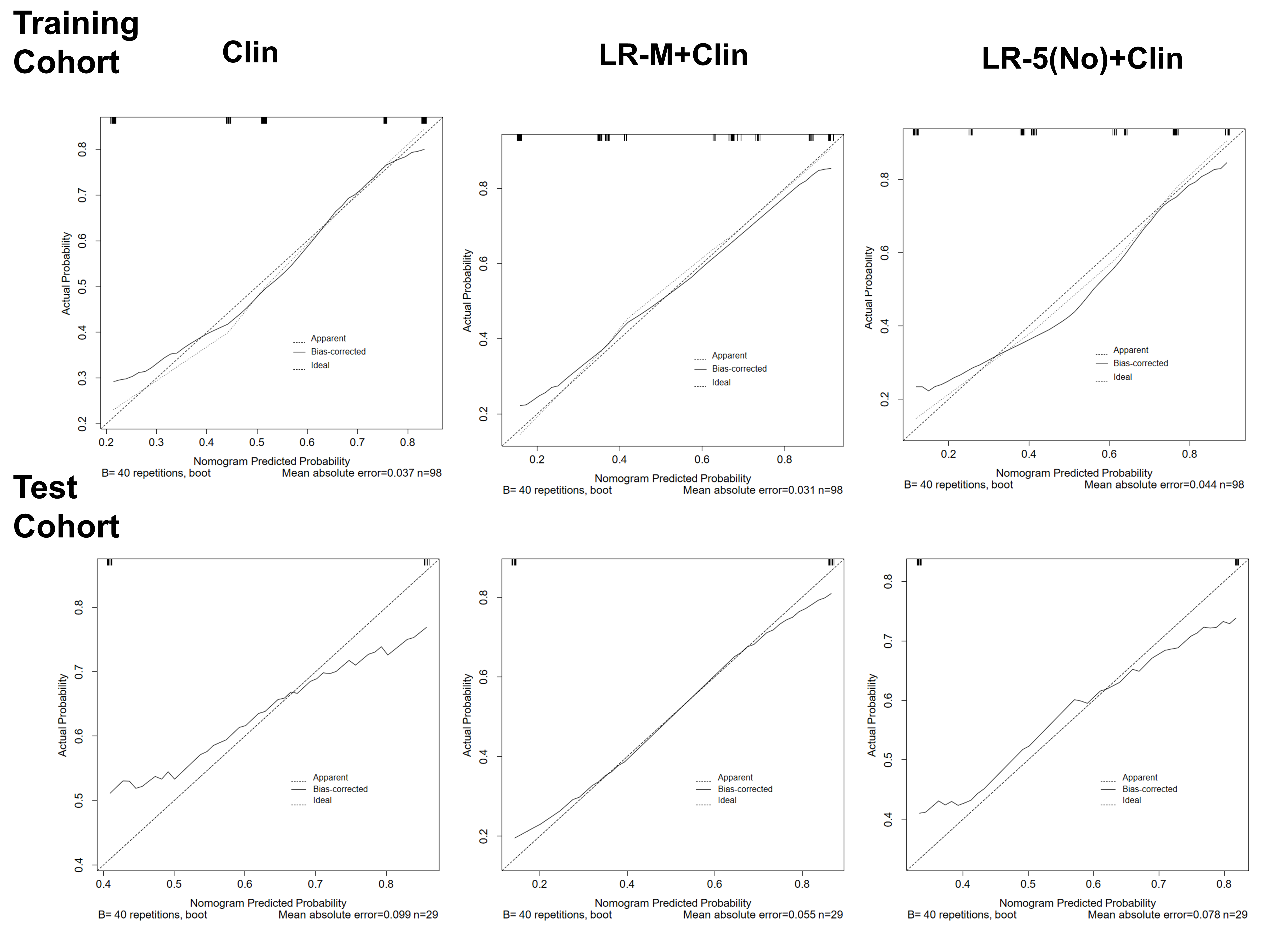

Supplement: Supplementary file 1 [file DataSheet_1.zip › 699200-raw data/calibration curve/calibration curve.tif]

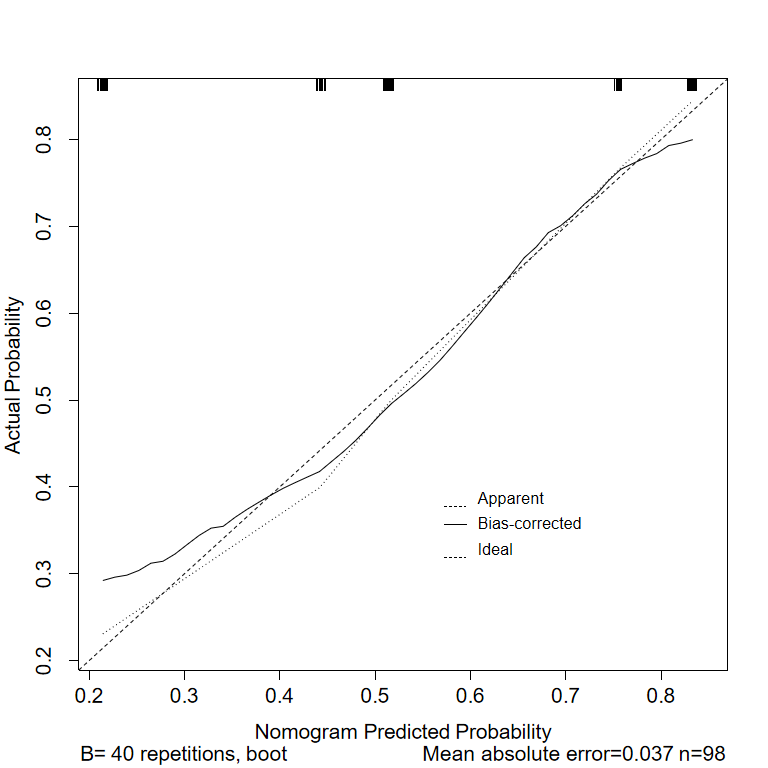

Supplement: Supplementary file 1 [file DataSheet_1.zip › 699200-raw data/calibration curve/train_clin_cali.tif]

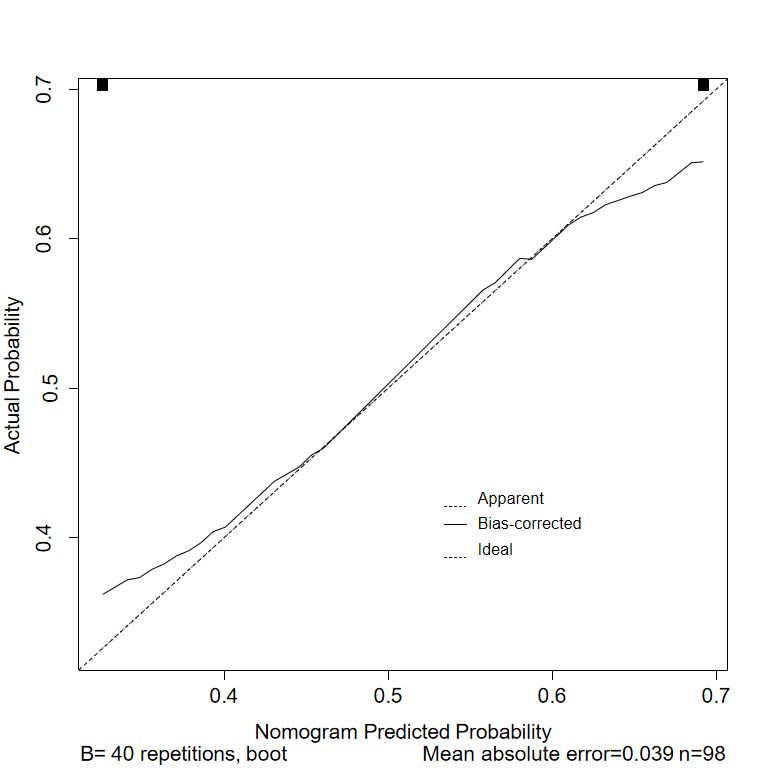

Supplement: Supplementary file 1 [file DataSheet_1.zip › 699200-raw data/calibration curve/train_lr5_cali.tif]

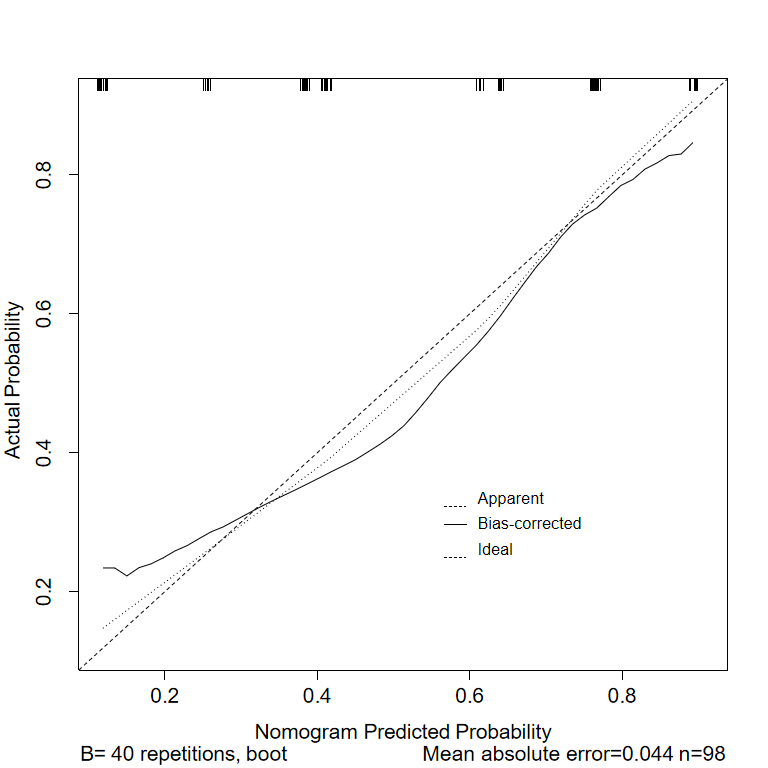

Supplement: Supplementary file 1 [file DataSheet_1.zip › 699200-raw data/calibration curve/train_lr5_clin_cali.tif]

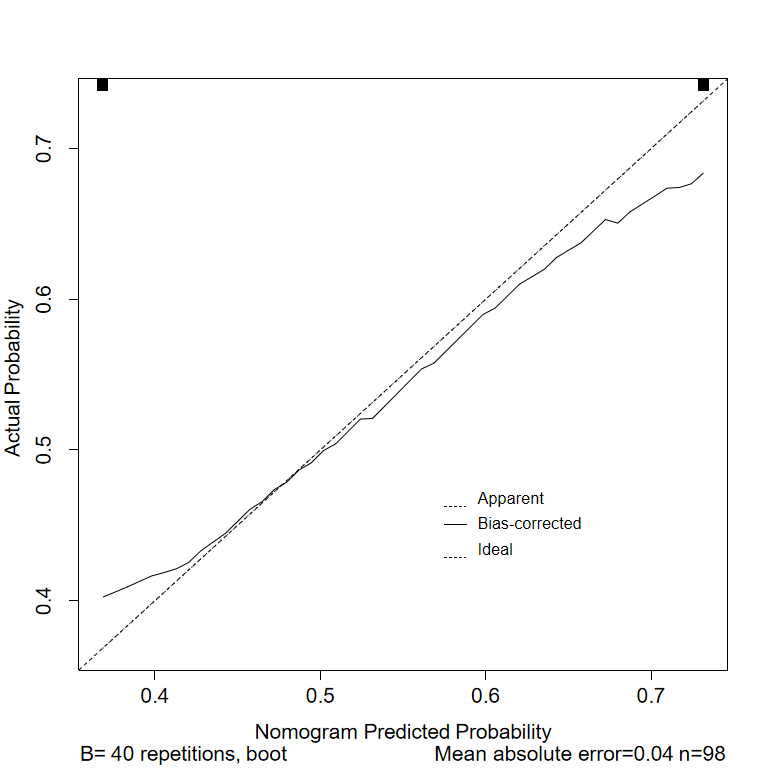

Supplement: Supplementary file 1 [file DataSheet_1.zip › 699200-raw data/calibration curve/train_lrm_cali.tif]

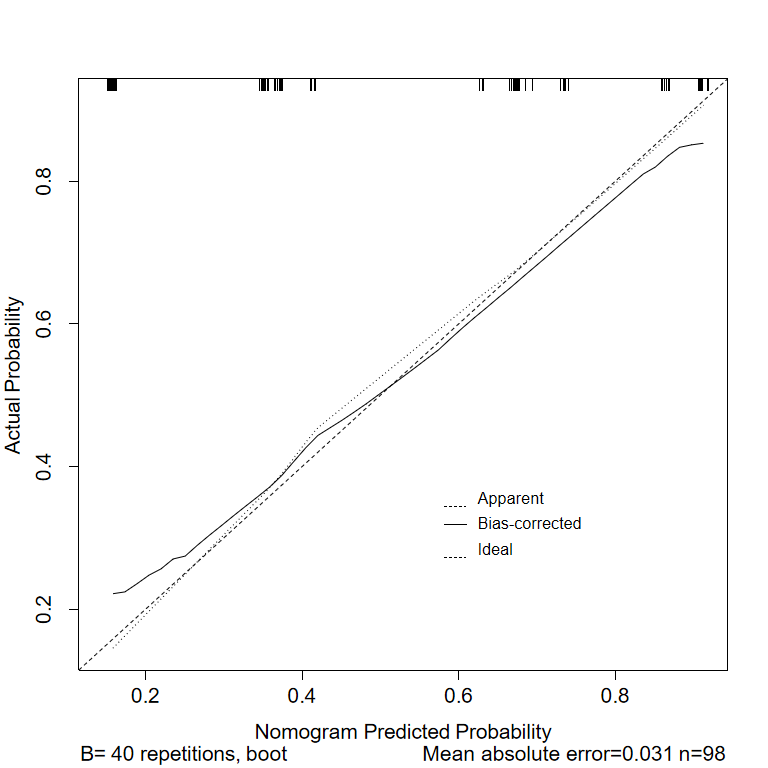

Supplement: Supplementary file 1 [file DataSheet_1.zip › 699200-raw data/calibration curve/train_lrm_clin_cali.tif]

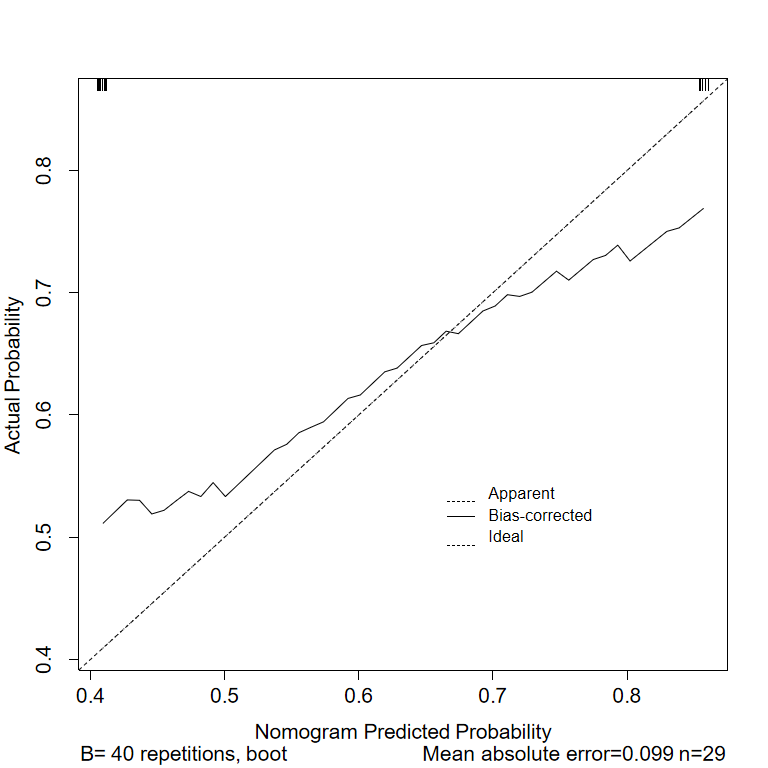

Supplement: Supplementary file 1 [file DataSheet_1.zip › 699200-raw data/calibration curve/valid_clin_cali.tif]

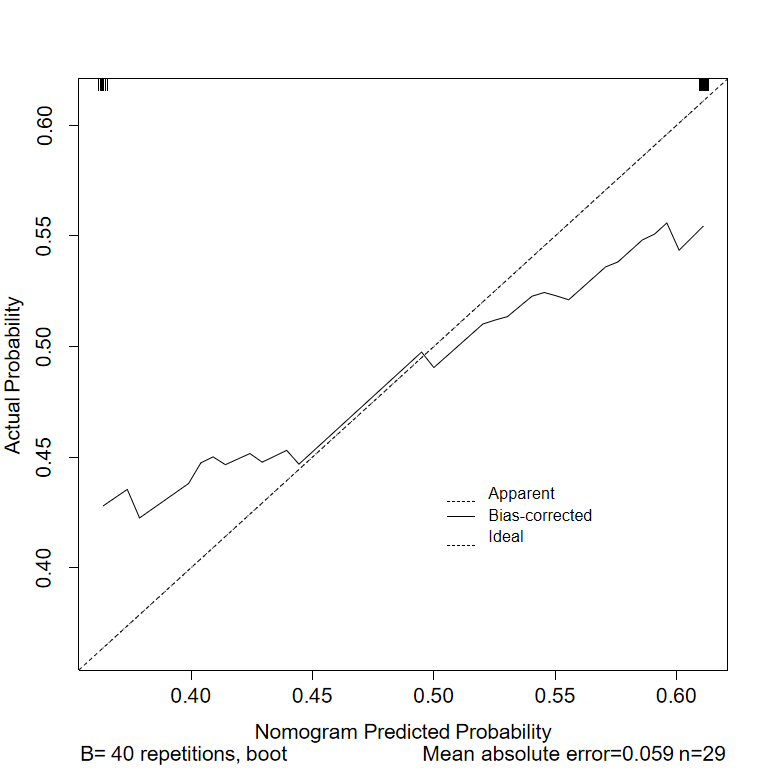

Supplement: Supplementary file 1 [file DataSheet_1.zip › 699200-raw data/calibration curve/valid_lr5_cali.tif]

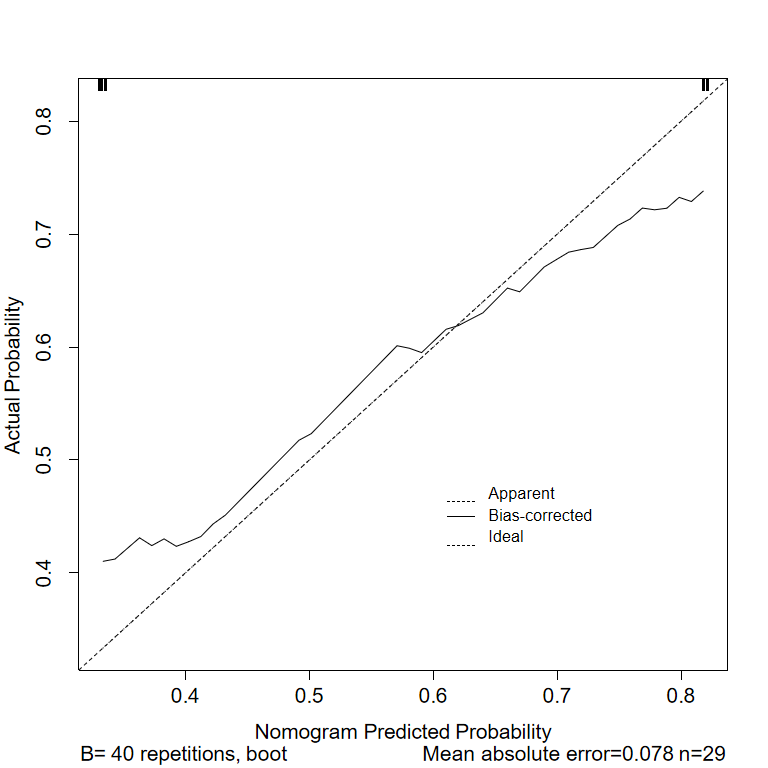

Supplement: Supplementary file 1 [file DataSheet_1.zip › 699200-raw data/calibration curve/valid_lr5_clin_cali.tif]

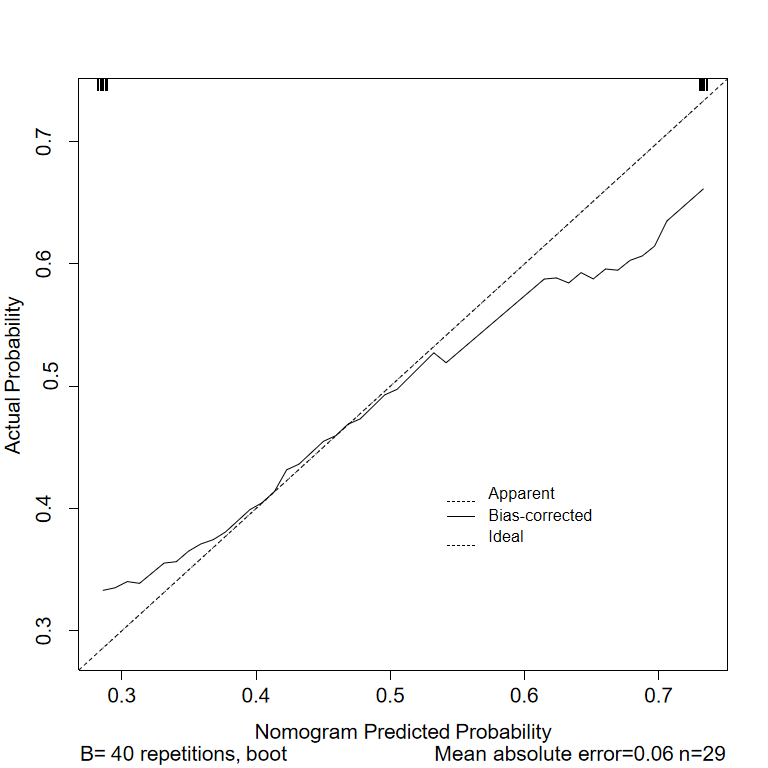

Supplement: Supplementary file 1 [file DataSheet_1.zip › 699200-raw data/calibration curve/valid_lrm_cali.tif]

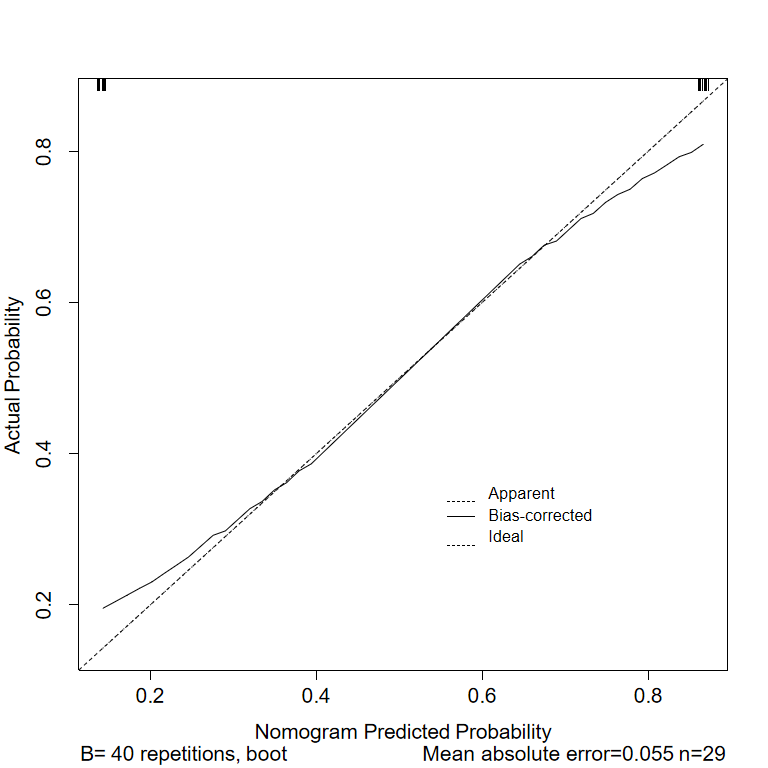

Supplement: Supplementary file 1 [file DataSheet_1.zip › 699200-raw data/calibration curve/valid_lrm_clin_cali.tif]

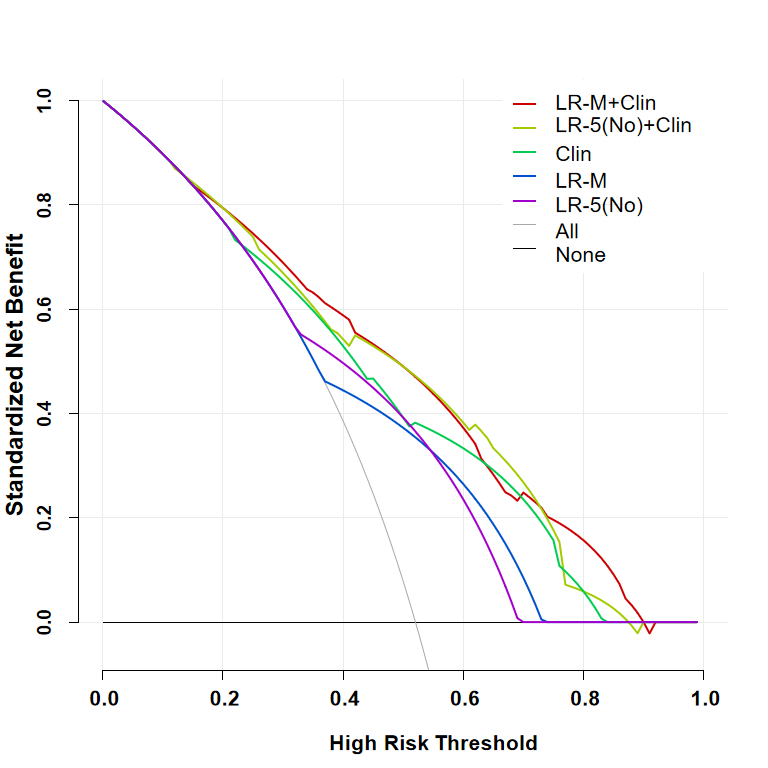

Supplement: Supplementary file 1 [file DataSheet_1.zip › 699200-raw data/decisioin curve/deci_curv_lr5_train.tif]

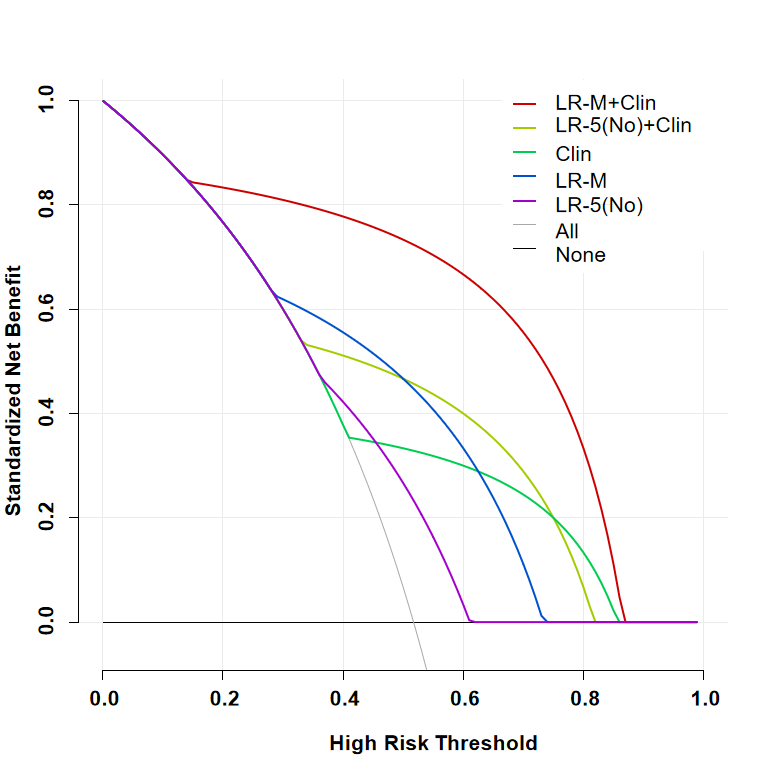

Supplement: Supplementary file 1 [file DataSheet_1.zip › 699200-raw data/decisioin curve/deci_curv_lr5_valid.tif]

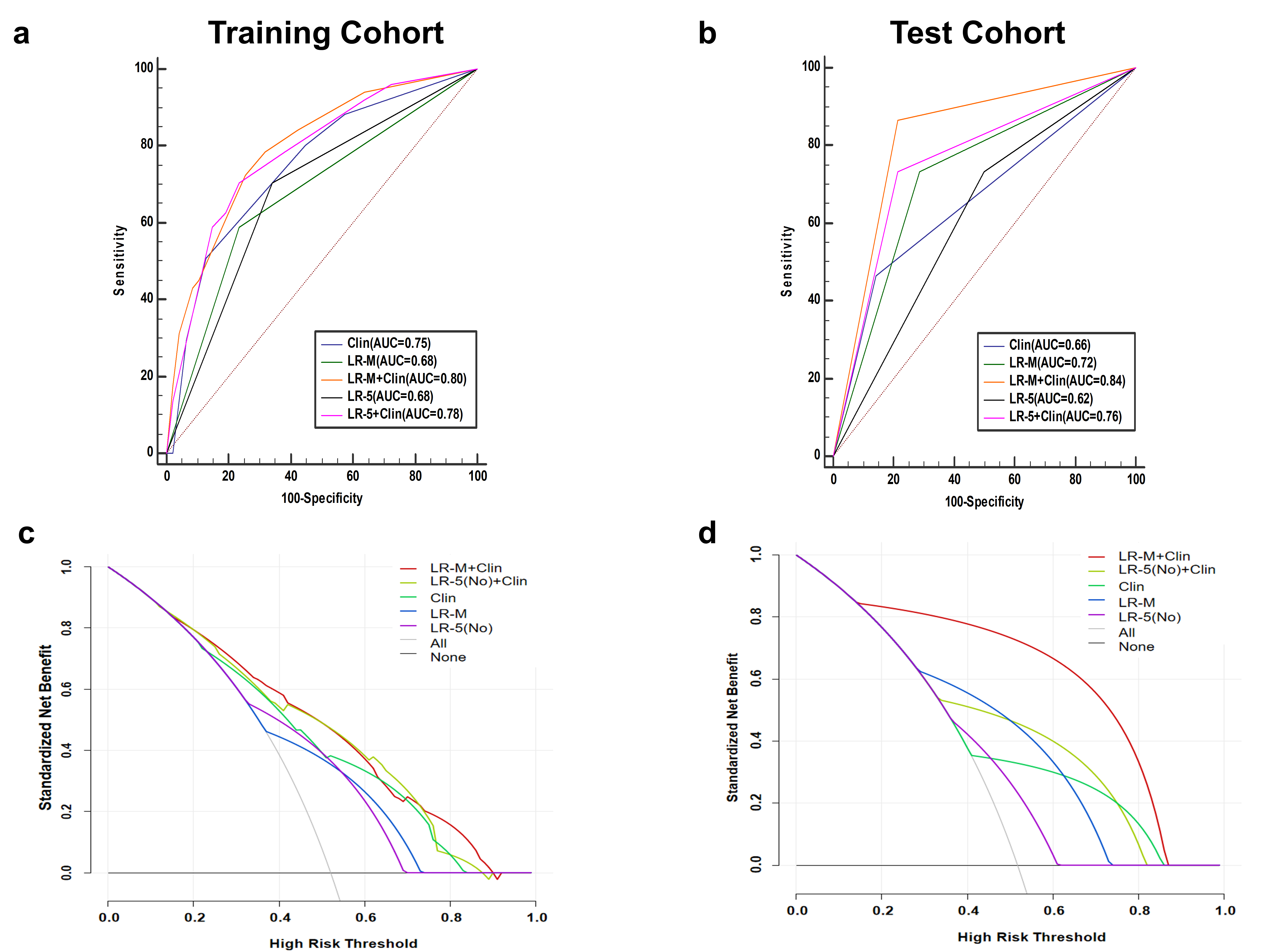

Supplement: Supplementary file 1 [file DataSheet_1.zip › 699200-raw data/decisioin curve/deicision curve+ROC.tif]

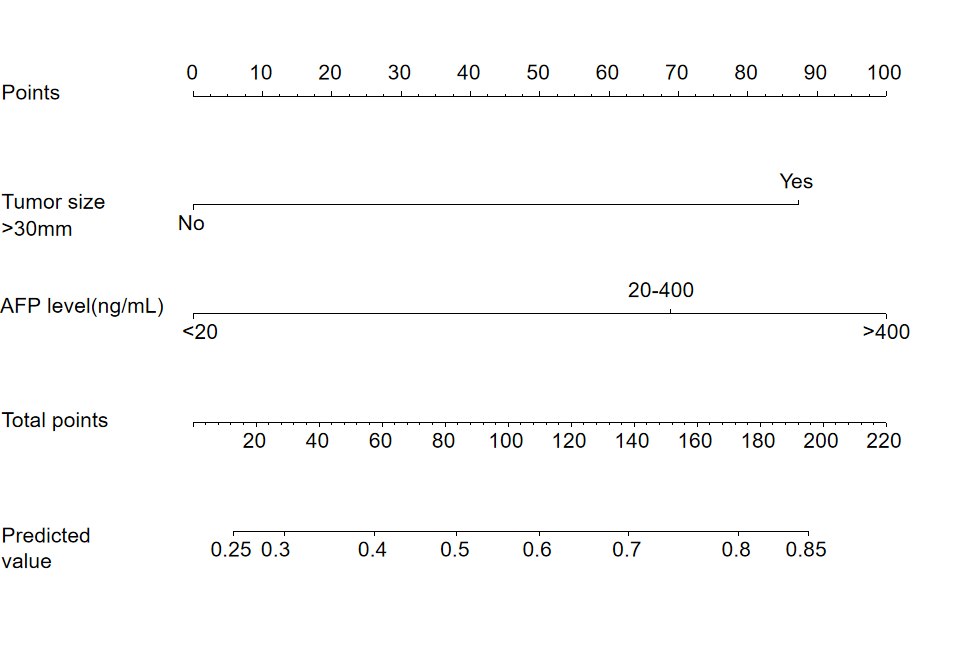

Supplement: Supplementary file 1 [file DataSheet_1.zip › 699200-raw data/nomogram figure/nom_clin.tif]

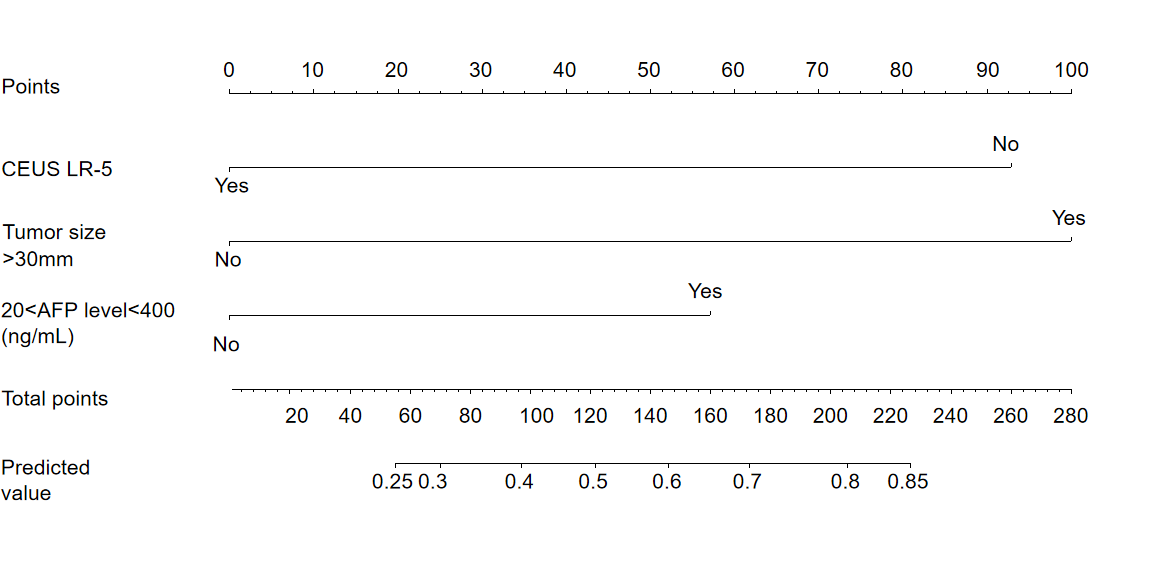

Supplement: Supplementary file 1 [file DataSheet_1.zip › 699200-raw data/nomogram figure/nom_lr5_clin.tif]

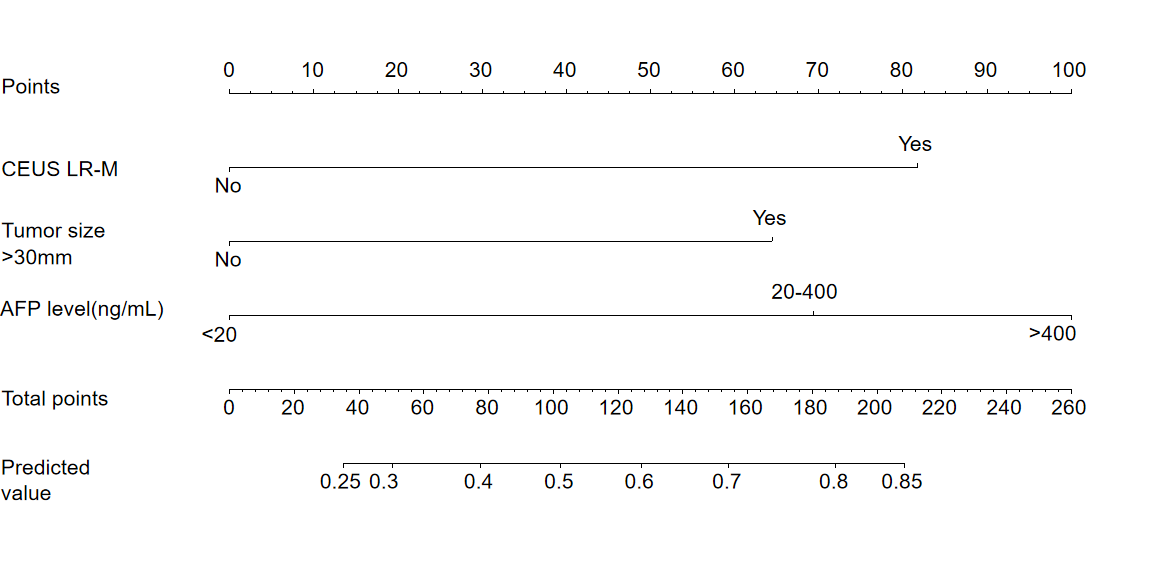

Supplement: Supplementary file 1 [file DataSheet_1.zip › 699200-raw data/nomogram figure/nom_lrm_clin.tif]

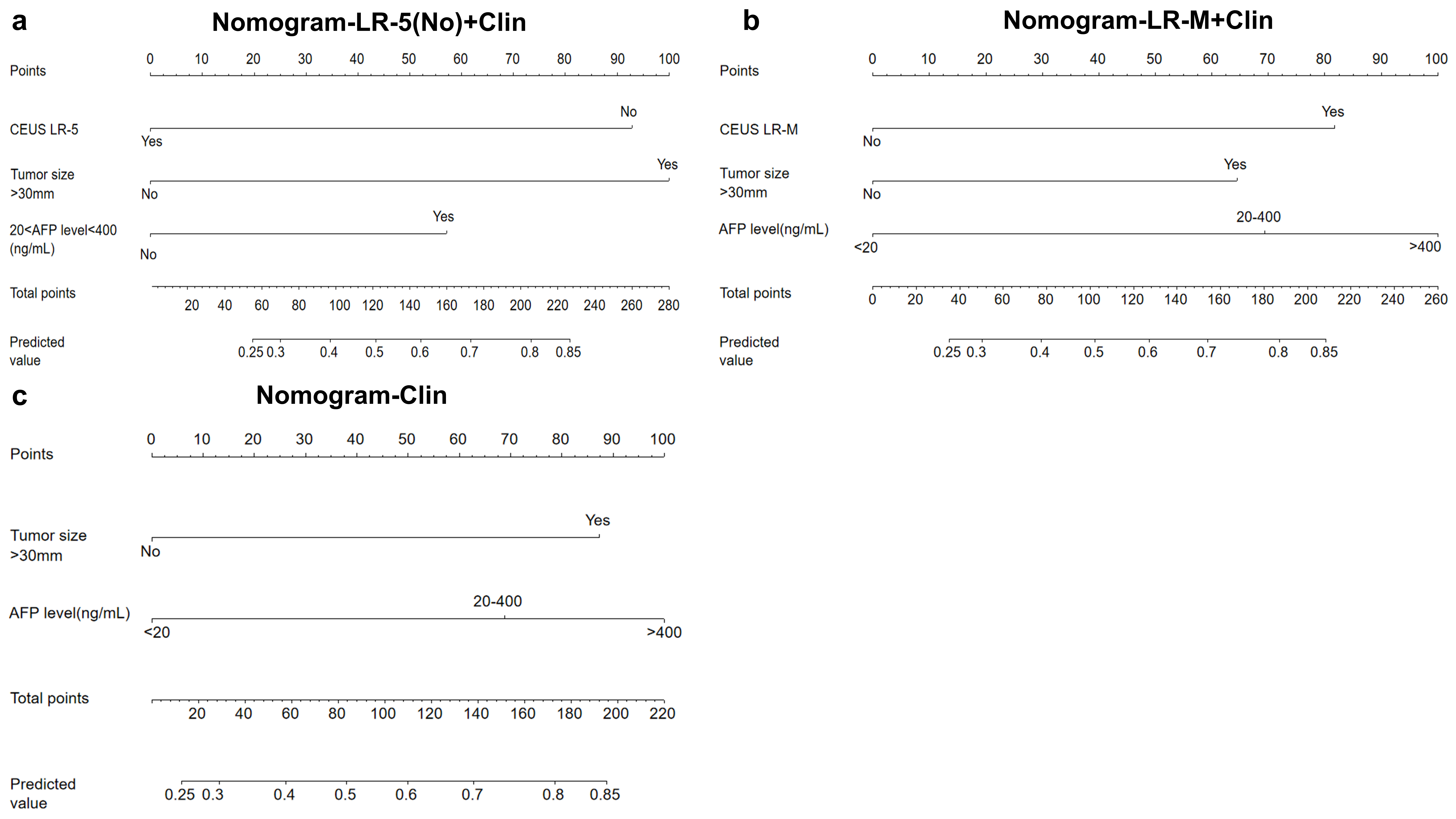

Supplement: Supplementary file 1 [file DataSheet_1.zip › 699200-raw data/nomogram figure/nomogram.tif]
