# Supplementary material for: A Nomogram Based on Combining Clinical Features and Contrast Enhanced Ultrasound LI-RADS Improves Prediction of Microvascular Invasion in Hepatocellular Carcinoma
Source: Front Oncol. 2021 Jul 8;11:699290. doi: 10.3389/fonc.2021.699290 (PMC8297520; doi:10.3389/fonc.2021.699290)
Supplement: Supplementary file 2 [file Table_1.doc]

**Supplemental information**

**Supplemental Table 1.** Definition of CEUS Imaging Features of FLL

| CEUS Feature | Definition |
| --- | --- |
| APHE  (not rim or peripheral discontinuous) | Enhancing part must be higher in echogenicity than liver in arterial phase, unequivocally greater in whole or in part than liver. |
| Late and mild washout | Temporally defined subtype of washout in which onset is beyond 60 seconds from contrast injection |
| Early Washout | Temporally defined subtype of washout in which onset is within 60 seconds from contrast injection |
| Marked Washout | Degree-defined subtype of washout in which the degree of washout is marked within 2 minutes after contrast injection |
| Rim APHE | Spatially defined subtype of APHE in which arterial phase enhancement is most pronounced in observation periphery |
| Nodule-in-nodule architecture | Presence of smaller inner nodule within and having different imaging features than larger outer nodule. In cirrhosis, suggests HCC |
| Mosaic architecture | Presence of randomly distributed internal nodules or compartments, usually with different imaging features |

Note: APHE, arterial phase hyperenhancement; CEUS, contrast-enhanced ultrasound; FLL, focal liver lesion

Supplemental Table 2. Basic information between training cohort and test cohort

|  | Training Cohort（n=98） | Test Cohort（n=29） | P value |
| --- | --- | --- | --- |
| Age | 46.4±33.6 | 39.1±26.8 | 0.288 |
| Male sex(yes) | 84(85.7%) | 25(86.2%) | 1.000 |
| Cirrhosis(yes) | 81(82.7%) | 20(69.0%) | 0.121 |
| Hepatitis B virus infection(yes) | 90(91.8%) | 24(82.8%) | 0.171 |
| PT>14s | 75(76.5%) | 22(75.9%) | 1.000 |
| TT>18s | 74(75.5%) | 22(75.9%) | 1.000 |
| AFP(<20/20-400/>400ng/mL) | 56/24/18 | 16/9/4 | 0.721 |
| Platelet count<100*10^9^ | 77(78.6%) | 21(72.4%) | 0.615 |
| Total bilirubin>21umol/L | 73(74.5%) | 25(86.2) | 0.218 |
| Albumin<35 | 22(22.4%) | 11(37.9%) | 0.095 |
| ALT>45U/L | 60(61.2%) | 21(72.4%) | 0.271 |
| AST>35U/L | 45(45.9%) | 17(58.6%) | 0.229 |
| Tumor size>30mm | 62(63.3%) | 15(51.7%) | 0.264 |

Note: ALT, Alanine aminotransferase; AST, Aspartate aminotransferase; AFP, alpha fetoprotein; PT, Prothrombin time, TT, thrombin time;

Supplemental Table 3. Univariate analysis of clinical, US and CEUS LI-RADS features for predicting MVI status in test cohort

|  | MVI Positive  (n=15) | MVI Negative  (n=14) | P value |
| --- | --- | --- | --- |
| Clinical features |  |  |  |
| Age | 57.1±9.1 | 58.4±9.2 | 0.692 |
| Male sex(yes) | 12(80.0%) | 13(92.9%) | 0.316 |
| Cirrhosis(yes) | 9(60.0%) | 11(78.6%) | 0.427 |
| Hepatitis B virus infection(yes) | 13(86.7%) | 11(78.6%) | 0.651 |
| PT>14s | 3(20.0%) | 4(28.6%) | 0.682 |
| TT>18s | 5(33.3%) | 2(14.3%) | 0.390 |
| AFP(<20/20-400/>400ng/mL) | 5/7/3 | 11/2/1 | 0.044 |
| Platelet count<100*10^9^ | 3(20.0%) | 5(35.7%) | 0.427 |
| Total bilirubin>21umol/L | 2(13.3%) | 2(14.3%) | 1.000 |
| Albumin<35 | 3(20.0%) | 5(35.7%) | 0.427 |
| ALT>45U/L | 2(13.3%) | 6(42.9%) | 0.109 |
| AST>35U/L | 6(40.0%) | 6(42.9%) | 1.000 |
| Tumor size>30mm | 11(73.3%) | 4(28.6%) | 0.027 |
| US features |  |  |  |
| Echogenicity（hypo/iso/hyper） | 10/0/5 | 9/3/2 | 0.116 |
| Poorly defined margin | 5 (33.3%) | 9 (64.3%) | 0.143 |
| Irregular shape | 6 (40.0%) | 11(78.6%) | 0.06 |
| Halo sign (yes) | 1(6.7%) | 2(14.3%) | 0.598 |
| Vascularity（no/a few/rich） | 8/6/1 | 2/10/2 | 0.086 |
| CEUS LI-RADS features |  |  |  |
| CEUS LR-5 major features |  |  |  |
| APHE (yes) | 12(80.0%) | 11(78.6%) | 1.000 |
| Late and mild WO (yes) | 5 (33.3%) | 9 (64.3%) | 0.143 |
| CEUS LR-M features |  |  |  |
| Rim enhancement (yes) | 2(13.3%) | 0 (0) | 0.483 |
| Early WO (yes) | 10 (66.3%) | 3 (21.4%) | 0.025 |
| Marked WO (yes) | 2(13.3%) | 1 (7.1%) | 1.000 |
| CEUS LI-RADS ancillary features |  |  |  |
| Nodule-in-nodule pattern (yes) | 1(6.7%) | 0 (0) | 1.000 |
| Mosaic pattern (yes) | 2(13.3%) | 0 (0) | 0.483 |
| CEUS LI-RADS(3/4/5/M) | 0/0/4/11 | 1/2/7/4 | 0.070 |
| CEUS LR-5 (No) | 11(73.3%) | 7(50.0%) | 0.264 |
| CEUS LR-M (yes) | 11(73.3%) | 4(28.6%) | 0.027 |

Note: ALT, Alanine aminotransferase; AST, Aspartate aminotransferase; AFP, alpha fetoprotein; PT, Prothrombin time, TT, thrombin time; CEUS, contrast-enhanced ultrasound; LI-RADS, liver imaging and reporting data system; APHE, arterial phase hyperenhancement; WO, washout
